# Epidermal keratinocytes regulate hyaluronan metabolism *via* extracellularly secreted hyaluronidase 1 and hyaluronan synthase 3

**DOI:** 10.1016/j.jbc.2024.107449

**Published:** 2024-06-04

**Authors:** Minori Abe, Manami Masuda, Yoichi Mizukami, Shintaro Inoue, Yukiko Mizutani

**Affiliations:** 1 Department of Cosmetic Health Science, Gifu Pharmaceutical University, Gifu, Japan; 2 Institute of Gene Research, Yamaguchi University Science Research Center, Ube, Yamaguchi, Japan

**Keywords:** hyaluronan, hyaluronic acid, hyaluronidase, keratinocyte, epidermis, metabolic regulation, cell differentiation, ultracentrifugation, ultraviolet-visible spectroscopy

## Abstract

Hyaluronan (HA) is a high-molecular-weight (HMW) glycosaminoglycan, which is a fundamental component of the extracellular matrix that is involved in a variety of biological processes. We previously showed that the HYBID/KIAA1199/CEMIP axis plays a key role in the depolymerization of HMW-HA in normal human dermal fibroblasts (NHDFs). However, its roles in normal human epidermal keratinocytes (NHEKs) remained unclear. HYBID mRNA expression in NHEKs was lower than that in NHDFs, and NHEKs showed no depolymerization of extracellular HMW-HA in culture, indicating that HYBID does not contribute to extracellular HA degradation. In this study, we found that the cell-free conditioned medium of NHEKs degraded HMW-HA under weakly acidic conditions (pH 4.8). This degrading activity was abolished by hyaluronidase 1 (HYAL1) knockdown but not by HYAL2 knockdown. Newly synthesized HYAL1 was mainly secreted extracellularly, and the secretion of HYAL1 was increased during differentiation, suggesting that epidermal interspace HA is physiologically degraded by HYAL1 according to pH decrease during stratum corneum formation. In HA synthesis, hyaluronan synthase 3 (HAS3) knockdown reduced HA production by NHEKs, and interferon-γ-dependent HA synthesis was correlated with increased HAS3 expression. Furthermore, HA production was increased by TMEM2 knockdown through enhanced HAS3 expression. These results indicate that NHEKs regulate HA metabolism *via* HYAL1 and HAS3, and TMEM2 is a regulator of HAS3-dependent HA production.

Hyaluronan (HA) is a non-sulfated linear glycosaminoglycan composed of repeating disaccharide units of N-acetyl-D-glucosamine (NAG) and D-glucuronic acid. It is widely distributed across various tissues and is an important component of the extracellular matrix. It has an average molecular mass of 4–6000 kDa. The skin contains more than 50% of the total body HA content (1, 2). The metabolism of HA is highly dynamic, and its half-life has been reported to be approximately 1 day in the skin (2, 3), 1 to 3 weeks in cartilage, and 2 to 5 min in blood (4, 5). It is well known that HA plays an important role in cell migration, inflammation, development, and cancer. Metabolic imbalance in HA greatly affects epidermal integrity by controlling cell proliferation and differentiation (6, 7). High-molecular-weight HA (HMW-HA; >1000 kDa) is responsible for the hydration and viscoelasticity of the skin and facilitates the transport of ion solutes and nutrients. It also exhibits anti-inflammatory and anti-angiogenic effects (8, 9). Studies on the naked mole-rat suggest that HMW-HA contributes to cancer resistance and longevity (10, 11). In contrast, low-molecular-weight HA (LMW-HA; <100 kDa) exhibits pro-inflammatory effects and enhances cancer cell invasion (12, 13). Thus, the balance between HA synthesis and depolymerization should be tightly regulated. However, it remains unclear what molecules are involved in HA metabolism in the skin, especially the epidermis.

Skin dermal fibroblasts play a crucial role in the degradation of HA in the dermis. We previously reported that HA-binding protein involved in HA depolymerization (HYBID/KIAA1199/CEMIP) plays a crucial role in HA-specific depolymerization in the dermis *via* normal human dermal fibroblasts (NHDFs) (14). HYBID plays a role in HA depolymerization independent of hyaluronidases (HYALs) and the cell surface HA receptor, CD44. The HYBID system utilizes clathrin-coated membrane regions, degrades incorporated HMW-HA (>1000 kDa) in early endosomes, and excretes HA molecules of intermediate size (10–100 kDa) into the extracellular spaces (14). Hyaluronidase transmembrane protein II (TMEM2/CEMIP2), which is reported to degrade HMW-HA into approximately 5 kDa fragments in a mouse model, was another candidate for dermal HA degradation (15). We reported that transforming growth factor-β (TGF-β), which suppresses HA depolymerization with a concomitant decrease in HYBID expression, upregulated TMEM2 in NHDFs (16). Additionally, human TMEM2 (hTMEM2) knockdown in NHDFs unexpectedly increased HYBID expression and enhanced HA depolymerization. These results suggest that hTMEM2 does not function as a hyaluronidase in NHDFs. Further, we also reported that HYAL1 and HYAL2/CD44 do not contribute to HA degradation in NHDFs (14).

On the other hand, in the epidermis, HA degradation mechanisms are not well understood. HMW-HA is abundant in the intercellular space, especially the basal layer in the epidermis (17, 18). The major candidates for HA degradation in the epidermis are HYAL1, HYAL2, HYBID, and TMEM2; however, their expression and HA degradation activity in epidermal keratinocytes remain unexplored. The HYAL1, acid-active endo-β-N acetyl hexosaminidase (optimum pH 3.8), degrades any size HA and is responsible for the final cleavage of HA into di- or tetra-saccharides (19, 20). The short half-life of HA in blood is attributed to receptor-mediated uptake of blood HA by liver sinusoidal endothelial cells, and internalized HA is rapidly degraded by HYAL1 (21). HYAL2 was initially reported to have an acidic optimum pH (22); however, recent studies revealed an optimum pH of 6.0 to 7.0, *in vitro* (23). Co-localization with CD44 on the cell surface is essential for the activity of HYAL2, which is responsible for degrading extracellular HA on the plasma membrane (23). A well-known classical HA degradation model (24) suggests that CD44 on the plasma membrane internalizes HMW-HA, followed by degradation to intermediate-sized HA in early endosomes by HYAL2, in tissues. Following this, HYAL1 degrades HA to oligosaccharides in the lysosomes. Although this model is promising for describing HA degradation in the liver, it is insufficient for understanding HA degradation in the skin.

Three isozymes of hyaluronan synthase (HAS) 1 to 3 exist in mammals. HASs on the plasma membrane are extremely fast-reacting enzymes and release newly synthesized HA to the cell exterior (25, 26, 27). The functional HAS varies, depending on the cell types and the regulation of epidermal HA synthase by HAS isozymes remains unknown. HAS2 is considered particularly important in mammals because HAS2 knockout mice show severe cardiac malformations (28). In the dermis, dermal fibroblasts are key for HA production. HAS2 is a major isozyme that synthesizes extracellular HA (29). Epidermal HA synthesis has been studied using rat epidermal keratinocytes, which revealed major upregulation of HAS2 expression by various factors such as EGF, KGF, and all-trans retinoic acid (6, 30, 31). In HaCaT cells (model for normal human keratinocytes), HAS2 was the major candidate for HA synthesis (32). However, when we used normal human epidermal keratinocytes (NHEKs), we detected HAS3 upregulation based on factors such as NAG and all-trans retinoic acid (33, 34, 35). Therefore, we proposed that HAS3 is key for HMW-HA synthesis in NHEKs. Considering the molecular distribution of HA in the stratum corneum (SC), it is necessary to distinguish whether LMW-HA in the SC exists due to degradation of HMW-HA or enhanced production of LMW-HA.

This study was aimed to elucidate the HA metabolic system in the epidermis. The mechanisms of HA depolymerization and the regulation of HA metabolism were investigated using NHEKs under various conditions. We revealed that, unlike the regulation of HA metabolism by the HYBID/HAS2/TMEM2 system in NHDFs, an extracellularly secreted HYAL1 and TMEM2-regulated HAS3 system is involved in the regulation of epidermal HA metabolism by NHEKs. This may help to elucidate and respond to pathophysiological changes in HA metabolism in the epidermis.

## Results

### HA degradation differed between human dermal fibroblasts (NHDFs) and human epidermal keratinocytes (NHEKs)

**Figure 1:**
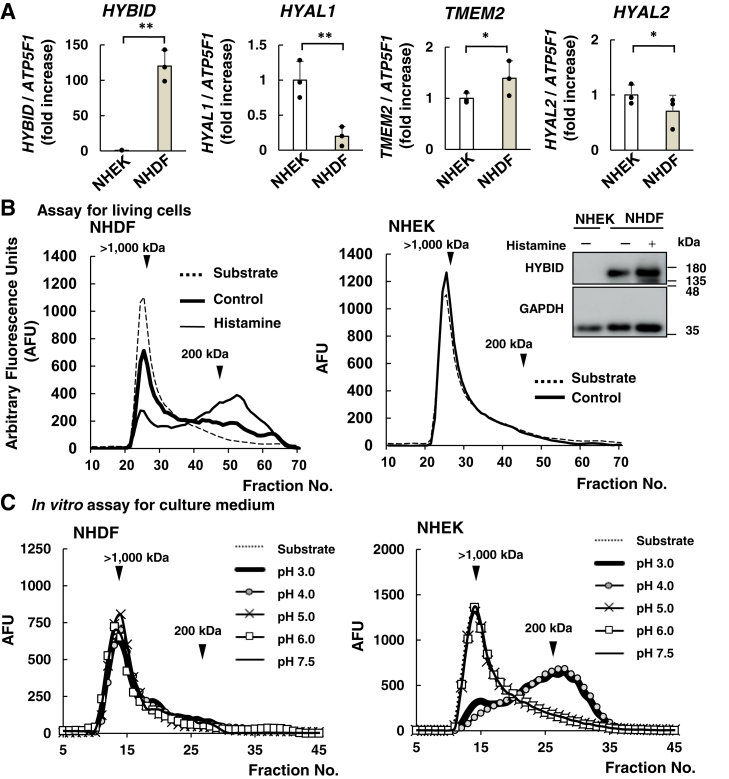
**HA degradation and expression of the related mRNA in NHEKs show patterns distinct from NHDFs**. *A*, relative expression of *HYBID*, *HYAL1*, *TMEM2*, and *HYAL2* mRNA were determined by RT-qPCR. Total RNA of NHEKs cultured in low-Ca^2+^ basal medium and NHDF cells were collected 1 day after reaching confluence. The expression levels were normalized using *ATP5F1* as the loading control. The expression of each target in NHEKs was set at 1.00 and the expression of NHDFs was shown as a ratio. Values represent the mean ± S.D.; n = 3; ∗*p* < 0.05, ∗∗*p* < 0.01 *versus* NHEKs (Dunnett’s test). *B*, HA depolymerization assay for living cells in NHEKs and NHDFs. To measure the HA-degrading activity of NHEKs and NHDFs, equal amounts of FITC-labeled HMW-HA were added to medium for 48 h. The resultant changes in molecular weight distribution of HA were measured by gel permeation chromatography, Sepharose CL-2B (0.7 × 50 cm). NHDFs were treated with or without histamine (10 μM). The positive control (Histamin+) revealed a peak shift to LMW-HA. The expression level of HYBID was detected *via* immunoblotting using an anti-HYBID antibody. GAPDH was used as the loading control. *C*, detection of HA degradation activity *in vitro* using culture medium at an acidic pH (3.0 and 4.0). The collected medium was assayed for the HA degradation *in vitro* under pH 3.0 to 7.5. The degraded FITC-HA fragments were analyzed using a Sepharose CL-2B column (0.7 × 30 cm). The substrate shows the original molecular size of FITC-HMW-HA (*dotted line*).

We compared the expression of hyaluronidases in NHDFs and NHEKs to clarify the differences in HA degradation mechanisms between the dermis and epidermis, respectively. The mRNA levels of HA depolymerization-related genes *HYBID*, *TMEM2*, and *HYAL*s were analyzed by quantitative reverse transcription PCR (RT-qPCR) in NHDFs and NHEKs. As shown in Figure 1*A*, *HYBID* mRNA expression was significantly high (*p* < 0.01) in NHDFs as compared with NHEKs, while the reverse was true in the case of *HYAL1* mRNA expression (*p* < 0.01). This trend was consistent with the next-generation sequencing results, which investigated the amount of RNA per million copies in each cell type (Table S1). Compared to the transcripts per million (TPM) in NHDFs, the TPM of *HYBID* and *HYAL1* in NHEKs was 0.02 and 9.5-fold greater, respectively.

To verify HA depolymerization in cultured cells, FITC-HMW-HA was added to the culture medium. In NHDF, HYBID-dependent HA fragments released from the cells by exocytosis into the extracellular medium were detected (Fig. 1*B* left). Histamine upregulated HYBID protein expression for up to 2 days in NHDFs (approximately 1.53-fold vs. without histamine). In NHDF, histamine-induced HYBID expression regulated an increase in FITC-HA depolymerization, which was indicated by an increase in the lower-molecular peak of intermediate-sized (10–100 kDa) fragment. We also examined the possible involvement of HYBID in epidermal HA degradation using NHEKs. *HYBID* mRNA expression in NHEKs was lower than that in NHDFs (Fig. 1*A*). A comparison of the relationship between HYBID protein expression and HA depolymerization activity in NHEKs and NHDFs did not reveal HYBID protein expression or increased extracellular HMW-HA depolymerization in NHEKs (Fig. 1*B* right). The siRNA-mediated downregulation of HYBID did not alter extracellular HMW-HA depolymerization in NHEKs (Fig. S1). These results suggested that HYBID is not a major contributor to extracellular HA degradation in NHEKs.

Further, we focused on the extracellular HA-degrading activity of NHEKs and examined pH-dependent HA degradation by extracellular proteins in cell-free conditioned culture medium of NHEKs and NHDFs *in vitro* using FITC-HMW-HA as a substrate. The optimal pH for hyaluronidase activity in the culture medium was determined using a wide pH range. At pH 3.0, 4.0, 5.0, 6.0, and 7.5, the culture medium was incubated with FITC-HMW-HA at 37 °C. Upon using the NHDFs culture medium, the molecular shift of FITC-HA was not detectable at any pH (Fig. 1*C*, left). However, upon using the NHEKs culture medium, the molecular shift in FITC-HA at pH 5.0 to 7.5 was undetectable, whereas FITC-HA degradation was detected at pH 3.0 and 4.0 (Fig. 1*C*, right). Therefore, HA depolymerization was regulated by different pathways in NHEKs and NHDFs.

### HYAL1 is a major target for HA degradation in NHEKs

**Figure 2:**
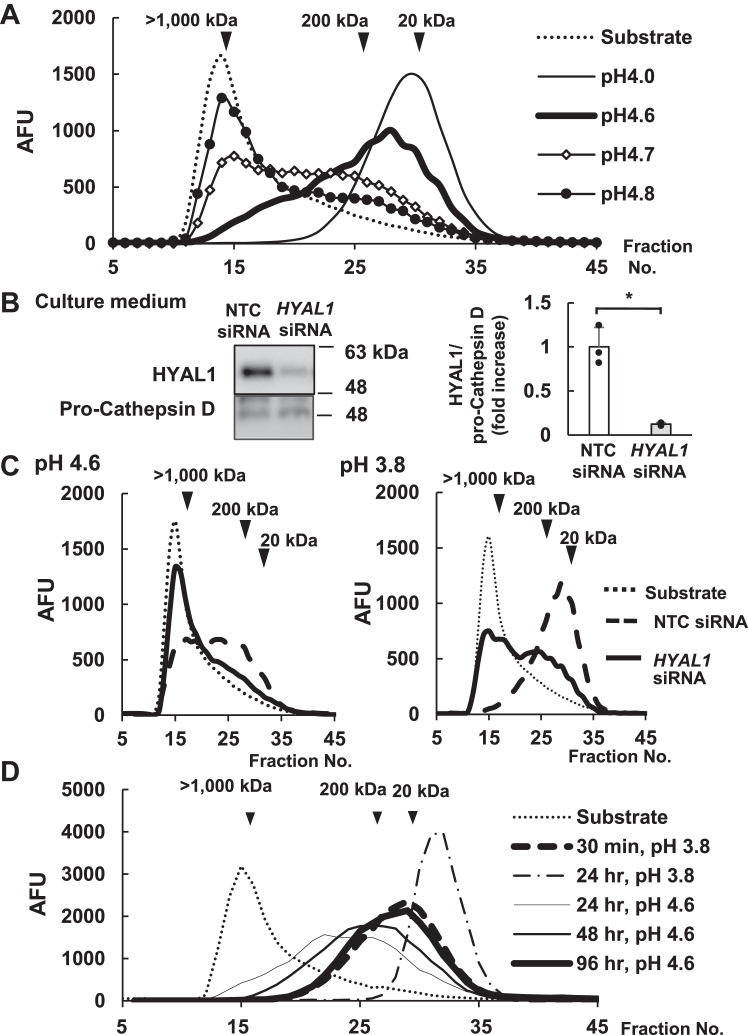
**HYAL1 shows HA degradation activity under weakly acidic conditions.***A*, HA depolymerization activity is detectable at an acidic pH under pH 4.8 *in vitro*. NHEKs were cultured in the high-Ca^2+^ differentiation medium for 3 days. The collected medium (from day 2 to 3) was assayed for HA degradation *in vitro* at pH 4.0 to 4.8. *B*, effects of *HYAL1* siRNA on protein expression in NHEKs. One day after the transfection of *HYAL1* siRNA, NHEK cells were cultured in the differentiation medium for 2 days. The protein expression of HYAL1 was detected using immunoblotting. The HYAL1 extracellular proteins are compared with the non-target control (NTC) or *HYAL1* siRNA. Cathepsin D is used as the loading control. The protein levels are shown as a fold increase relative to control (NTC siRNA) expressed as means ± S.D. (n = 3). ∗*p* < 0.05 *versus* the NTC siRNA (Student’s *t* test). *C*, *HYAL1* siRNA reduced the molecular shift in HA depolymerization. The cultured medium from *HYAL1* or NTC siRNA was adjusted to an *in vitro* assay at pH 4.6 and 3.8. *D*, comparison of pH-dependent reaction rate *in vitro*. A culture medium of differentiated NHEKs was used for HA degradation assay *in vitro.* Degradation activity was compared between optimum pH (pH 3.8, 30 min, and 24 h) and pH 4.6 (24, 48, 96 h). FITC-HA fragments were determined on a Sepharose CL-2B column (0.7 × 30 cm). The substrate shows the original molecular size of FITC-HMW-HA (*thin dotted line*). Three independent experiments were performed. NTC, non-target control.

Based on Figure 1*C*, in an *in vitro* assay using NHEKs culture medium, degraded HA was detected around fraction No. 28 under acidic conditions. To gain additional information, HA decomposition was observed at pH 4.0, 4.6, 4.7, and 4.8 using a high Ca^2+^ differentiation culture medium (Fig. 2*A*). The molecular shift in FITC-HA was clearly observed in fraction number 25–35 at pH 4.0. At pH 4.6, all HMW-HA shifted to the LMW side. Additionally, hyaluronidase activity at pH 4.7 and 4.8 was quite low and the shift from the substrate fractions was partial. Hyaluronidase activity was undetectable at pH 5.0 and the peak fractions were similar to those of the substrate fractions (Fig. 1*C*, right). Thus, the lower limit for detection of HA degradation was pH 4.8 (Fig. 2*A*).

In the epidermis, HMW-HA condenses in the intercellular spaces with an acidic physiological pH ranging from 4.1 to 5.8 in the SC and upper epidermis (36). Our results showed that the cell-free conditioned medium of NHEKs degraded HMW-HA under acidic conditions and the expression of *HYAL1* (optimum pH of 3.8) mRNA in NHEKs was higher than that in NHDFs (Fig. 1). Based on the hypothesis that HYAL1 degrades HMW-HA in the intercellular space of the epidermis, we used *HYAL1* siRNA to confirm its HA-degrading activity. HYAL1 knockdown in NHEKs was confirmed by detecting protein expression levels in the culture medium by immunoblotting. Under this condition, the production of extracellular HYAL1 protein in the culture medium was inhibited by *HYAL1* siRNA by approximately 87.6% (Fig. 2*B*). Pro-cathepsin D is a control protein released by Ca^2+^ stimulation in NHEKs; however, its expression was not altered by *HYAL1* siRNA. The optimum pH of HYAL1 is 3.8 and the human epidermal SC shows an acidic pH. Thus, we set pH 4.6 and 3.8 as the conditions for this *in vitro* assay of the culture medium. Results revealed that *HYAL1* siRNA blocks HA degradation at pH 4.6 and 3.8 *in vitro* (Fig. 2*C*). We compared the reaction rate between pH 4.6 and 3.8 *in vitro*. At pH 3.8, we detected the molecular shift in HMW-HA towards around 20 kDa in 30 min and to the end of fractions in 24 h. In contrast, at pH 4.6, 96 h was necessary to observe the same degradation reaction as in 30 min at pH 3.8 (Fig. 2*D*). We also examined the effect of HYAL2 expression in cell-free *in vitro* assay. Based on next-generation sequencing, *HYAL2* mRNA expression in NHEKs was 10-fold higher than that of HYAL1 (Table S1). Nevertheless, *HYAL2* siRNA did not affect HA degradation at pH 4.6 *in vitro* (Fig. S2).

**Figure 3:**
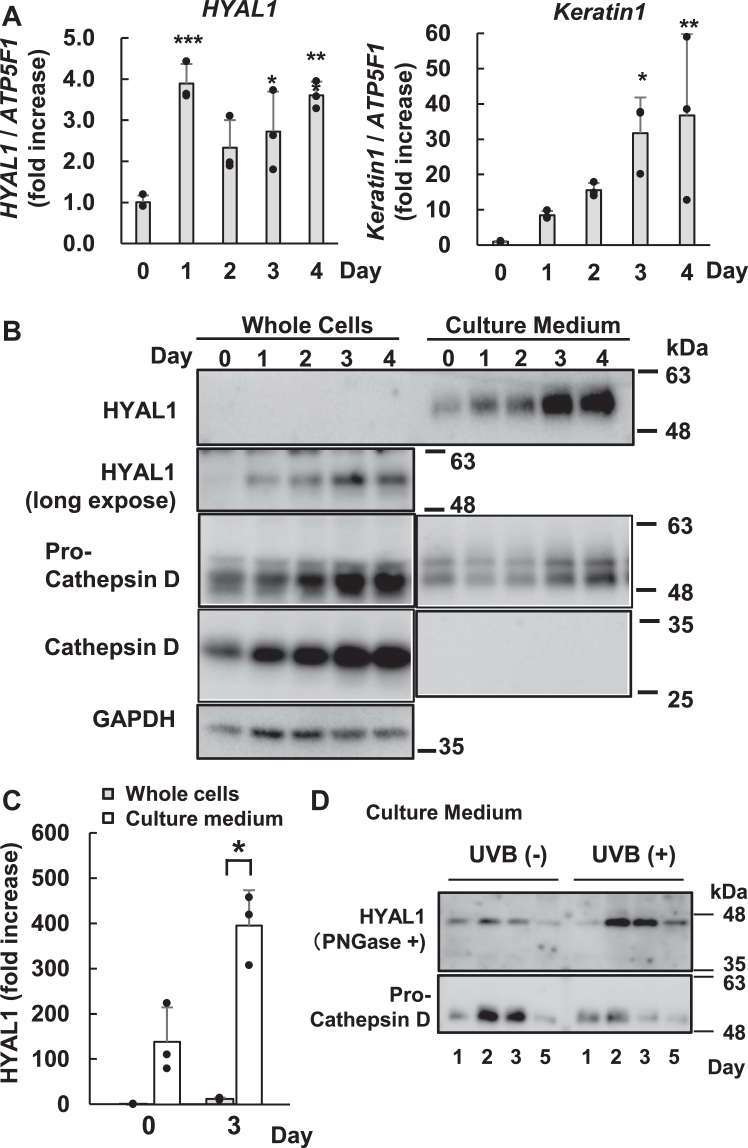
**Differentiation of NHEKs induced the upregulation and extracellular release of HYAL1**. NHEK cells were cultured in the differentiation medium for 4 days (*A*) The mRNA expression levels of *HYAL1* and *Keratin1* (differentiation marker) were determined by RT-qPCR. The expression levels were normalized by *ATP5F1*, which was used as a loading control. Values represent the mean ± S.D. (n = 3) ∗*p* < 0.05, ∗∗*p* < 0.01, ∗∗∗*p* < 0.005 *versus* the control (day 0) (Dunnett’s *t* test). *B*, intracellular (*left*, whole cells) and extracellular (*right*, culture medium) HYAL1 protein expression during differentiation detected by anti-HYAL1 antibody. HYAL1 in culture medium was detected after a short exposure time (*upper panel*, 10 s) and whole cells were detected after long exposure (*lower panel*, 1 min). Anti-cathepsin D antibodies detected both pro-cathepsin D and mature cathepsin D. GAPDH was used as a loading control. *C*, HYAL1 protein expression between whole cells and culture medium was compared and shown as a ratio using immunoblotting. The whole cells (0 days) were set at 1.0 and the fold change in HYAL1 amount is shown as the ratio. Values represent the mean ± S.D. (n = 3) ∗*p* < 0.05 *versus* each whole cell (Student’s *t* test). *D*, HYAL1 and pro-cathepsin D in the culture medium were detected by immunoblotting in differentiated NHEKs induced by UVB irradiation (30 mJ/cm^2^). Following PNGase F treatment, HYAL1 was detected as a single band (∼45 kDa). Non-irradiated cells were used as the negative control.

Further, we examined the relation between the upregulation of HYAL1 and HA degradation in NHEKs. During differentiation, NHEKs changed their protein expression, including differentiation markers. Under differentiating conditions, *HYAL1* mRNA expression increased from day 1, and differentiation marker *Keratin 1* mRNA showed a time-dependent increase from day 1 to 4 (RT-qPCR data) (Fig. 3*A*). Following differentiation, we detected an increase in HYAL1 expression through immunoblotting and compared the expression in whole cells and the culture medium (Fig. 3*B*). HYAL1 expression and release increased during differentiation. The highest concentration of extracellular proteins was detected on day 4. In addition, compared to the extracellular release of HYAL1, the intracellular level of this protein was quite low and required longer exposure for detection (Fig. 3*B*). The amount of HYAL1 in the culture medium was more abundant than that in whole cells (Fig. 3*C*). As a comparison, we examined the extracellular release of pro-cathepsin D (∼46 kDa) and mature cathepsin D (∼28 kDa), which matures in the lysosome, for comparison (Fig. 3*B*). Both forms were elevated in whole cells during differentiation. In the culture medium, pro cathepsin D was detected; however, the mature form was undetectable. UVB induces dynamic HA depolymerization 2 days post-irradiation in mice (37). Therefore, we examined the upregulation of released HYAL1 in UVB-stimulated NHEKs. At 30 mJ/cm^2^, extracellularly secreted HYAL1 was upregulated during NHEK differentiation (Fig. 3*D*). The extracellular secretion of pro-cathepsin D was not upregulated by UVB irradiation.

**Figure 4:**
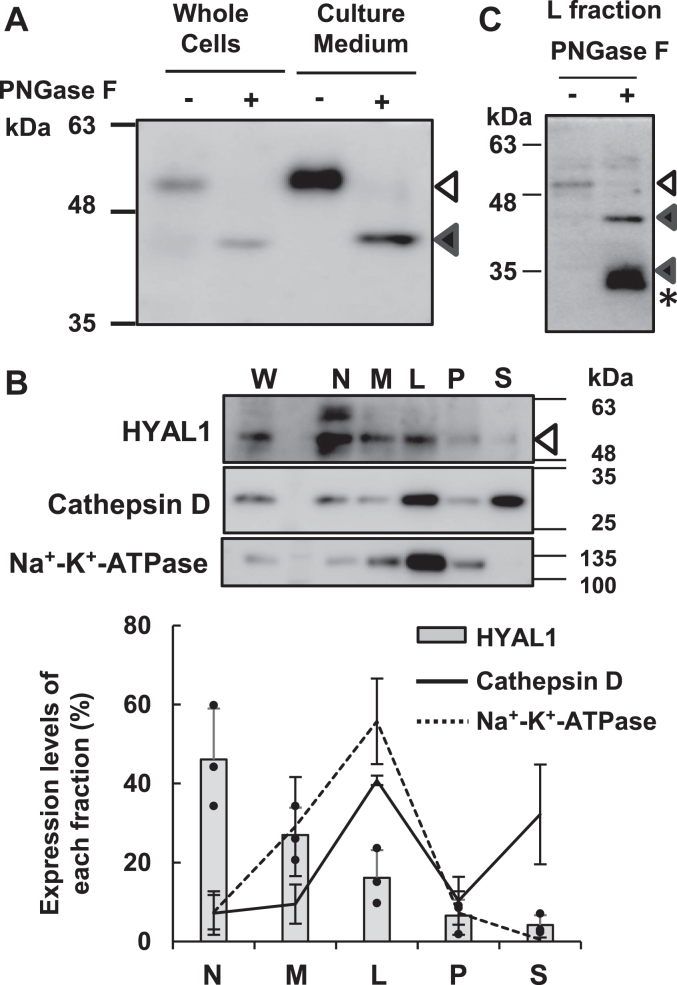
**Full-length HYAL1 is present in the culture medium**. NHEKs were cultured in a high Ca^2+^ differentiation medium. *A*, the cleaved form of HYAL1 was not detected in the culture medium. The whole cells and culture medium on day 3 were treated with PNGase F (200 units) to investigate the deglycosylated forms. The glycosylation form (*open arrow*) of HYAL1 was changed to the deglycosylated form (*closed arrows*). Following PNGase F treatment, the full length was detected as a major band (∼45 kDa), and the cleaved form of HYAL1 (∼37 kDa), which is reported to cleave in lysosome, could not detected. *B*, detection of HYAL1, mature Cathepsin D (lysosomal marker), and Na⁺-K⁺-ATPase (cell membrane marker) content of N, M, L, P, and S fractions obtained from whole cell fraction of NHEKs, by immunoblotting. The ratio of each protein expressed in fractions is shown as a %. The total amount of W was 100%. The expression level of each band was quantified, and the values represent the mean ± S.D. (n = 3). The average was calculated from three independent experiments. *C*, the cleaved form of HYAL1 was detected in the L fraction. The L fraction was treated with or without PNGase F (200 units). Following PNGase F treatment, the glycosylation form (*open arrow*) of HYAL1 changed to the deglycosylated form (*closed arrows*). The full length (∼45 kDa) and the cleaved form (∼37 kDa) were detected. L, light mitochondrial fraction; M, heavy mitochondrial fraction; N, nuclear fraction; P, microsomal fraction; and S, cytosolic fraction.

We detected the molecular size of HYAL1 using PNGase F (removes all N-linked glycans) treatment to examine the extracellular trafficking pathway of HYAL1 in NHEKs. Newly synthesized HYAL1 is glycosylated in the Golgi apparatus, and HYAL1 translocated to the lysosome is cleaved at the lysosome. Before treatment with PNGase F, the molecular size of HYAL1 in whole cells and culture medium was the same. After treatment, only the expression of non-cleaved (full-length pre-lysosomal) HYAL1 (∼45 kDa) was detected in the culture medium of NHEK (Fig. 4*A*). In whole-cell homogenates, HYAL1 cleaved in the lysosome (∼37 kDa) was undetectable, and non-cleaved HYAL1 was detected as a major band. To study the trafficking of HYAL1 to the lysosome, we prepared a nuclear fraction (N), heavy mitochondrial fraction (M), light mitochondrial fraction (L), microsomal fraction (P), and cytosolic fraction (S). M and L fractions are predicted lysosome fractions. In this condition, a major cathepsin D peak (mature form in lysosomes) was exhibited in the L fraction. Na⁺-K⁺-ATPase, a cell membrane marker, was mainly found in the L fraction. HYAL1 is partially localized in the M and L fractions (Fig. 4*B*). After treating the L fraction with PNGase, cleaved HYAL1 (∼37 kDa) was detectable (Fig. 4*C*). These results suggest that newly synthesized HYAL1 was released extracellularly directly from the Golgi apparatus.

### HMW-HA production in NHEKs is regulated by HAS3

**Figure 5:**
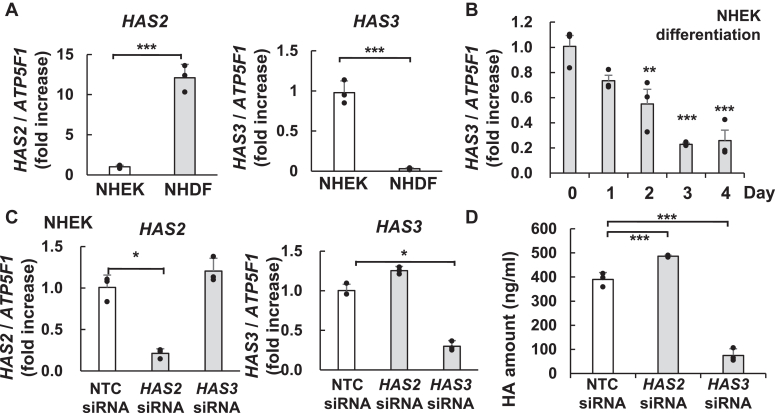
**HA synthase, HAS3 is the major enzyme for HMW-HA synthesis in NHEKs.***A*, *HAS2* and *HAS3* mRNA expression levels determined by qRT-PCR. Total RNA was the same as in Figure 1*A*. The expression levels were normalized using *ATP5F1* as the loading control. The expression of each target in NHEK cells is set at 1.00 and the expression level of NHDF is shown as a ratio. *B*, *HAS3* mRNA expression levels were downregulated during differentiation. Total RNA was the same as in Figure 3*A*. The expression level in undifferentiated NHEKs (day 0) is set at 1.00. *C*, relative mRNA expression of *HAS2* and *HAS3* in NHEKs as detected by qRT-PCR. Following transfection with *HAS2* or *HAS3* siRNA, NHEKs was cultured in a basal medium for 4 days (*D*) The amount of HA in each medium (day 3–4) was detected using the HA Quantification Kit. Values represent the mean ± S.D. (n = 3). ∗*p* < 0.05, ∗∗*p* < 0.01, ∗∗∗*p* < 0.005 *versus* the Control (NTC siRNA) (Dunnett’s *t* test).

To identify the factors responsible for the production of HMW-HA in NHEKs, we measured the mRNA expression levels of HASs. Table S1 shows HAS3 as the major transcript of the HA synthase family in NHEKs. The TPM value of *HAS3* mRNA expression was the highest in NHEKs, that is, 43- and 23-fold that in *HAS1* and *HAS2*, respectively. However, in NHDFs, HAS2 was responsible for HA synthesis and the TPM of *HAS2* was the highest. While the TPM value of *HAS2* in NHEKs was approximately 0.4 times that of NHDFs, *HAS3* showed a 79.5-fold higher TPM value in NHEKs compared to NHDFs (Table S1). Furthermore, RT-qPCR analysis confirmed a similar trend (Fig. 5*A*) with higher expression of *HAS3* than *HAS2* in NHEKs. No *HAS1* mRNA was detected through RT-qPCR under our experimental conditions. As HMW-HA is abundant in the basal layer of the epidermis (17, 18), we detected downregulated *HAS3* mRNA expression during differentiation in NHEKs (Fig. 5*B*). However, a previous report suggested that HAS2 is a potent enzyme for the synthesis of HMW-HA and that HAS3 is a medium-molecular-weight HA (MMW-HA) synthase *in vitro* (38). To determine whether HAS2 or HAS3 is important for HMW-HA synthesis in NHEK cells, we knocked down *HAS3* or *HAS2* in NHEKs using appropriate siRNAs for gene silencing for 2 days. The relative amounts of mRNA transcripts were assessed to confirm the specific downregulation of *HAS2* mRNA (85%) and *HAS3* mRNA (74%) using RT-qPCR (Fig. 5*C*). We examined HA accumulation in the culture medium with or without *HAS2* or *HAS3* siRNA. Accumulation of HA was inhibited by approximately 81% after *HAS3* siRNA treatment, whereas *HAS2* siRNA treatment had no effect (Fig. 5*D*).

**Figure 6:**
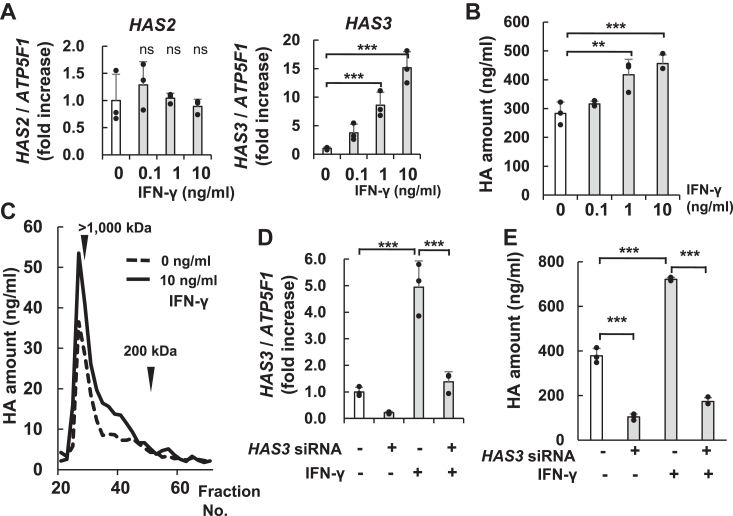
**IFN-γ induced HAS3 expression and HMW-HA synthesis in NHEKs.***A*, mRNA levels of *HAS2* and *HAS3* were detected by RT-qPCR after IFN-γ (0, 0.1, 1, 10 ng/ml) induction for 6 h. *B*, detection of HA amount in the medium after induction with IFN-γ for 24 h. *C*, molecular size distribution of HA after NHEKs stimulation with or without IFN-γ (10 ng/ml) for 24 h. *D*, The *HAS3* mRNA level after transfection with NTC siRNA or *HAS3* siRNA and cultured with or without IFN-γ (10 ng/ml) for 6 h was detected by RT-qPCR. *E*, the concentration of HA in the medium, following transfection of NHEKs with NTC or *HAS3* siRNA and culturing them for 24 h. The mRNA expression levels were normalized by *ATP5F1.* Values represent the mean ± S.D. (n = 3). ∗∗*p* < 0.01, ∗∗∗*p* < 0.005 *versus* the control (nontreated) (Dunnett’s test or Tukey’s test). ns, not significant.

The role of HAS3 in HMW-HA synthesis was evaluated using Interferon (IFN)-γ, known to enhance HA production in NHEKs (33). Its dose-dependent effects on HAS expression were examined. As shown in Figure 6*A*, the dose-dependent effect on *HAS3* mRNA expression was the highest at 10 ng/ml of IFN-γ, and expression was 15-fold higher as compared with that in the untreated control. In contrast, no dose-dependent changes were observed in *HAS2* mRNA expression. HA concentration in the medium also increased in the presence of IFN-γ in a dose-dependent manner. Along with HAS3 upregulation, IFN-γ concentrations of 1 to 10 ng/ml increased the accumulation of synthesized HA in the culture medium (Fig. 6*B*). Further, the molecular weight distribution of HA with and without IFN-γ stimulation in the culture medium was analyzed. The culture supernatant was fractionated by gel filtration chromatography, after 24 h of IFN-γ stimulation, and the concentration of HA in each fraction was evaluated. The synthesized HA in the NHEK culture medium had HMW-HA, approximately >1000 kDa, and the top peak (fraction number 26) showed a 1.5-fold increase as compared with that in the untreated control (Fig. 6*C*). In the growth medium, IFN-γ specifically increased the number of HA chains over a large size range, >1000 kDa. Although it increased the total amount of HA, it did not change the molecular weight distribution (Fig. S3*A*). These results revealed that HAS3 was majorly responsible for the induction of HA synthesis and increased the concentration of HMW-HA in NHEKs.

Furthermore, the increase in HA production due to HAS3 expression was confirmed by stimulating *HAS3* siRNA-treated cells with IFN-γ (10 ng/ml). RT-qPCR results showed that after 6 h, IFN-γ induced *HAS3* mRNA expression, which was suppressed by *HAS3* siRNA (Fig. 6*D*). HAS3 was mainly expressed in NHEKs monolayer cultures. After 24 h, the HA concentration increased 1.9-fold by IFN-γ stimulation, but this increase was suppressed by *HAS3* siRNA treatment (Fig. 6*E*). This result suggests that IFN-γ-induced HAS3 produces HMW-HA in NHEK cultures. The upregulation of HAS3 was mainly responsible for the induction of HA synthesis and increased the amount of HA in the medium.

**Figure 7:**
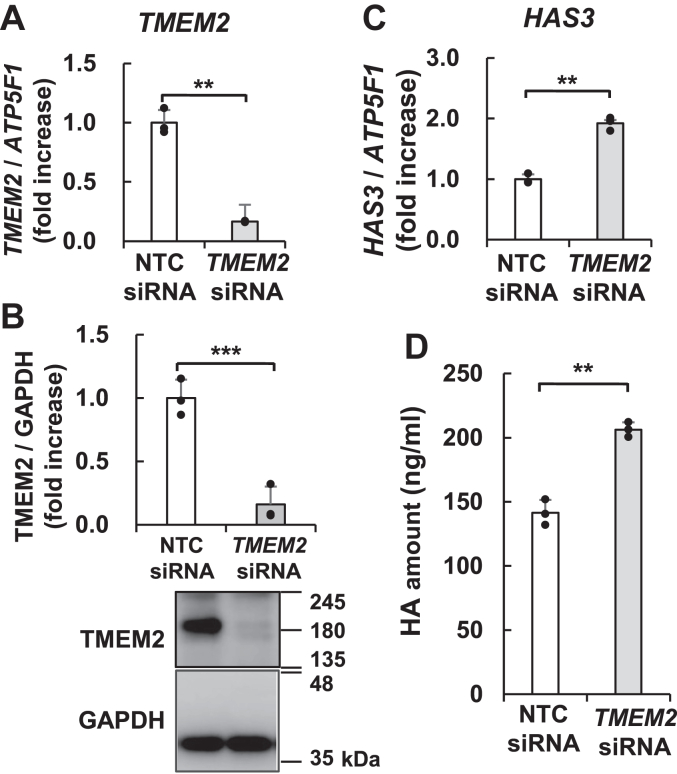
**TMEM2 regulates HAS3 expression and HA synthesis in NHEKs.** Following transfection with *TMEM2* siRNA, NHEKs were cultured in a basal medium. The samples were collected after 1 day. The mRNA expression of *TMEM2* (*A*) and *HAS3* (*C*) was determined by RT-qPCR. The mRNA expression levels are normalized to *ATP5F1*. *B*, TMEM2 expression, as detected by immunoblotting using an anti-TMEM2 antibody. GAPDH was used as a loading control. *D*, HA concentration in the culture medium. The HA concentration in each medium was detected using the HA Quantification Kit. Values represent the mean ± S.D. (n = 3). ∗∗*p* < 0.01, ∗∗∗*p* < 0.005 *versus* the control (NTC siRNA) (Student’s *t* test).

Next, we examined whether human TMEM2 is involved in the regulation of HA metabolism in NHEKs similar to NHDFs. After treatment with *TMEM2* siRNA, the expression of *TMEM2* mRNA was suppressed by 84%, and TMEM2 production decreased by 85% compared to the control (Fig. 7, *A* and *B*). Simultaneously, *HAS3* mRNA expression increased by 1.9-fold, and the amount of HA produced by the cells increased by 1.5-fold (Fig. 7, *C* and *D*). On the other hand, the downregulation of *HYAL1* did not alter the expression of *HAS3* mRNA (Fig. S4). Based on these results, we demonstrate the possibility of regulating HA synthesis *via* TMEM2 in NHEKs.

## Discussion

In human skin, the regulatory mechanism of HA metabolism appears to differ between the epidermis and dermis. In the dermis: HMW-HA, which is mainly produced by HAS2, is depolymerized by the HYBID endocytosis system of NHDFs. Depolymerized HA is released extracellularly and transported to the bloodstream or lymphatic system (14). However, in the epidermis, blood and lymph vessels are present only immediately below the basement membrane. Thus, the biosynthesis and degradation of HA in the epidermis essentially depend on the constituent cells, NHEKs. In this study, we propose a mechanism for HA metabolism in the epidermis using NHEKs as follows: HYAL1 is upregulated during differentiation, and its extracellular release results in the degradation of HMW-HA, which is mainly produced by HAS3, in the intercellular spaces.

In HYAL1 knock-outs, HA in the SC, which is negligible in wild-type (WT) mice, accumulates as MMW- and HMW-HA (39). The immunostaining of human skin revealed that HYAL1 was mainly distributed in the viable epidermal layers (40) and the SC above the granular layer (39). HA staining was stronger in the intercellular space of NHEKs below the spinous layer. The degradation of HMW-HA at an acidic pH was detected in human skin lysates, *in vitro* (41). The abundance of LMW-HA in normal SC (42) suggests that HMW-HA in intercellular space is degraded during differentiation. We considered the possibility that HYAL1 is released extracellularly in NHEKs, constituents of the epidermis, and can degrade HMW-HA in the intercellular spaces, where HA is present at concentrations as high as 2 mg/ml (1). In the epidermis, the pH of the basal layer is nearly neutral, whereas that of the SC is 4.1 to 5.5 in healthy individuals (43). Acidic conditions in the skin are also important for the production of ceramides, which are involved in the barrier function of SC. The exocytosis of lamellar bodies maintains the pH at 4.5 to 5.0 in the lower part of SC (44). However, pH changes lead to decreased activity of acid sphingomyelinase and beta-glucocerebrosidase, which inhibit the formation of intercellular lipid membranes in the SC (45). HYAL1 is an endo-type enzyme for all-size HA. At optimum pH, *i.e.*, around 3.8, HYAL1 reacts rapidly for the final cleavage of HA into di- or tetra-saccharides. However, it has been reported that HYAL1 prefers longer substrates at pH 5.5 (46). Our experiments confirmed that HMW-HA depolymerizes *in vitro* at pH 4.8 or lower, and the reaction rate of hyaluronidases at a slightly acidic pH of 4.6 is much lower than that of HYAL1 at the optimal pH and does not reach final cleavage (Fig. 2*D*). These results suggest that HYAL1 depolymerizes HMW-HA but does not reach final cleavage under weakly acidic conditions.

The co-localization of HYAL1 with the lysosomal protein Cathepsin D has been detected in granule cells immediately below the SC using immunostaining (39). We also detected partial HYAL1 colocalization in the lysosomal fraction, which shows major localization of mature Cathepsin D, after NHEKs differentiation (Fig. 4*B*). However, the addition of FITC-HMW-HA to the differentiation medium resulted in 97 to 99% recovery of HMW-HA after 2 days. If there is strong intracellular HYAL1 activity in the lysosome, FITC-HMW-HA added to the medium should be detected as LMW-HA after a few days, and a lack of strong activity should be seen as a decrease in the amount of HMW-HA in the medium. However, HMW-HA did not show any significant molecular changes in the medium even after a long period (approximately 4 days) in the culture environment of NHEKs, where the pH was neutral. This result suggests that the classical HA degradation (24), which internalizes HMW-HA as a substrate followed by lysosomal HYAL1-mediated HA depolymerization, is weak in NHEK cells.

Common acid-hydrolyzing enzymes undergo mannose-6-phosphate modification in the Golgi apparatus after recognition by Man-6P receptors, followed by transfer to the lysosomes. However, HYAL1 has not been detected in the Man-6-P lysosomal proteome (47). In macrophages, glycosylated HYAL1 is released extracellularly *via* endoplasmic reticulum transport and is subsequently taken up in the lysosome *via* mannose receptor-dependent endocytosis (48). In osteoclasts, HYAL1 is secreted through lysosomal exocytosis and constitutive secretion promotes the extracellular residency of HYAL1 through the downregulation of the mannose receptor (49). Osteoclasts express a vacuolar-type proton pump H+-ATPase on the plasma membrane, which releases protons to the extracellular space, acidifying the bone surface (50). These reactions would also help identify the conditions that promote an extracellular accumulation of HYAL1 and create acidic conditions in the intercellular spaces of multilayered NHEKs in the epidermis. The HMW-HA in the intercellular spaces may be degraded *via* the formation of acidic compartments on the plasma membrane during differentiation. Further studies are required to determine the regulatory mechanisms involved in maintaining the condensation to create acidic conditions in intercellular areas during epidermal differentiation.

Žádníková, P. *et al.* proposed that HYAL2 comprised the main HA-degrading enzyme in the skin (41). They show that in an *in vitro* assay of lysate from the human epidermis under acidic conditions, HMW-HA resulted in the production of intermediate-size fragments around 20 kDa (the end product size of HYAL2), which is different from the end product of HYAL1. The general HA-degrading activity of HYAL2 is much lower than that of HYAL1; however, the expression level of HYAL2 in NHEKs is relatively high. Immunostaining for HYAL2 in skin tissue shows its extended-expression throughout the epidermal layer (40). HA content in the ear skin of HYAL2 knockout mice was reported to be 3-fold higher than that in WT mice. The function of HYAL2 in human skin cannot be ignored, given the high expression of *HYAL2* mRNA in NHEKs. In our study, HYAL2-dependent HA-degrading activity was not detected in living cells or *in vitro*. However, *in vitro* experiments have limitations in demonstrating the contribution of HYAL2 in the epidermis. The contribution of HYAL2 to extracellular HA degradation requires further investigation, including the involvement of co-factors such as CD44 on plasma membranes (23, 51).

Immunohistological staining revealed that HYBID is expressed in the basal layer of the epidermis as well as in the dermal fibroblast (41). When extracellular HMW-HA is endocytosed by HYBID, the depolymerized HA (10–100 kDa) is released extracellularly (16, 52). We previously reported HA degradation in mouse epidermis under conditions of UV irradiation (37). Recently, it has been reported that HYBID protein expression in the human epidermis is enhanced following UV irradiation (53). An analysis of HA degradation-related gene expression showed that HYBID has the potential to increase HA degradation in NHEKs under UV irradiation (Fig. S5). However, NHEKs did not show extracellular HMW-HA degradation activity under this *HYBID* mRNA upregulated condition. IFN-γ stimulation increased *HYBID* mRNA expression but did not change the molecular weight distribution of HA in the culture medium (Fig. S3). These results indicate that HYBID is unlikely to play a central role in HMW-HA metabolism in NHEKs. Our results suggest that increased expression of *HYBID* mRNA by stimulation does not directly lead to meaningful HA degradation in terms of physiological function. However, some undetectable functional units may be involved in HYBID-dependent HA degradation. Zhang W *et al.* suggest that the enzymatic activity of HYBID is necessary to bind with ANAX1 at the G8 domain (54). HYBID may possess enzymatic activity under suitable conditions or in the presence of essential cofactors. If the expression and localization of the subunits together with HYBID are consistent, HYBID involvement in HMW-HA depolymerization only under specific conditions in the epidermis could be possible. Additionally, we suggest that hTMEM2 does not act in the depolymerization of extracellular HMW-HA in NHEKs. Yamamoto *et al.* showed that recombinant mouse TMEM2 (mTMEM2) degrades HMW-HA to approximately 5 kDa fragments using the HEK293T cell overexpression system (15). However, we reported that hTMEM2 does not exhibit HA-degrading activity in the overexpression system of HEK293T cells (55). Niu *et al.* also reported that hTMEM2 ectodomain is at least 10 ^7^-fold less active than that of conventional HYALs, because of its weak interaction with HA (56). These results reveal that the regulatory target genes of HA degradation in the epidermis are completely different from those in the dermis.

In addition, we also show that the enzymatic pathway and regulation of HA synthesis differ between the dermis and the epidermis. In NHDFs, HAS2 is the major HMW-HA synthase (57). Contrarily, here we demonstrated that HAS3, not HAS2, is the major enzyme for HMW-HA synthesis in NHEKs. In a recent study, siRNA-mediated knockdown of HAS3 in an epidermal equivalent model resulted in a significant reduction in epidermal HA content and thickness (58). Inflamed skin has an increased accumulation of HA due to increased *HAS3* mRNA expression in the affected areas of acute eczema (59). These studies support our hypothesis. Consistent with previous reports, a dose-dependent increase in HAS3 upregulation by inflammatory cytokines such as interleukin (IL)-4, IL-13, and IFN-γ, and by keratinocyte growth factor in NHEKs was observed (6, 30, 33, 60). In conclusion, all of the above evidence indicates that HAS3 is the major HMW-HA synthase in the NHEKs.

HA synthesis is also independently regulated in NHEKs and NHDFs by multiple factors. In NHEKs, TGF-β stimulation induced a decrease in HA production owing to HAS3 downregulation (33). Opposite responses to HAS2 upregulation-dependent HA synthesis have been reported in NHDFs (29). NAG is a rate-limiting substrate for HA synthesis and shows a dose-dependent increase in HA production in NHEKs but not in NHDFs (34). We propose that hTMEM2, unlike mTMEM2, does not function as a cell surface hyaluronidase but instead acts as the regulator of HA metabolism. This may result from the differences within the intracellular domain (ICD) of human and mouse TMEM2. A clear α-helical structure is observed only in human TMEM2. A binding site for the SH3 domain and a nuclear migration motif (KQKRHK) were also detected. These findings suggest that the hTMEM2 ICD may regulate intracellular signaling pathways (55). Interestingly, the regulatory mechanisms of HA metabolism by hTMEM2 also differ between the NHEKs and NHDFs. We previously showed that TGF-β1 suppressed HA degradation by decreasing HYBID expression, conversely promoting hTMEM2 expression in NHDFs (16). Another study showed that a decrease in HYBID expression by IL-1β and TGF-β1 was reversed by hTMEM2 knockdown (55). These results show that hTMEM2 is a regulator of HA metabolism in NHDF through *HYBID* and *HAS2* expression. Consequently, we also found the involvement of hTMEM2 in the regulation of HA metabolism in NHEKs; wherein hTMEM2 knockdown increased HA synthesis by upregulating HAS3 (Fig. 7). From these results, the expression of hTMEM2 in NHDFs and NHEKs induced the opposite reaction to HA accumulation through the regulation of gene expressions. The regulation mechanism of HA turnover in the epidermis and dermis is not yet understood. Further studies are necessary to elucidate the regulation of HA metabolism-related gene expression, including focusing on the ICD of hTMEM2.

In this study, we particularly focused on the enzymes involved in HA degradation and synthesis and showed that they differ between NHEKs and NHDFs. The limitation of this study is that we only employed an *in vitro* system for our investigations. However, tissue staining of HA in the skin suggests that HA is rapidly degraded in the granular layer immediately below the SC (17, 18). We propose a mechanism whereby active HMW-HA production by HAS3 occurs in the undifferentiated basal layer of NHEKs, after which HYAL1 is released during differentiation and accumulated in the intercellular spaces with HMW-HA, while the depolymerization of intercellular HMW-HA occurs in the layers above the tight junction of the granular layer. The absolute amount and molecular size of HA in the skin must be balanced to maintain its health. It was revealed that not only did the metabolic enzymes in the epidermis and dermis differ, but their reaction to regulators also showed differences. This makes it possible to utilize independent target-specific approaches for the maintenance of HA molecular weight distribution in the dermis and epidermis. The elucidation of HMW-HA formation and its degradation in the skin paves the way for studying new pharmacological targets to introduce innovative ideas in dermatology, orthopedics, and pathophysiology.

## Experimental procedures

### Cell culture and stimulation

NHEKs, procured from Lifeline Cell Technology (KK-4009, Frederick), were used to obtain a serum-free primary culture model. NHEKs were maintained in MCDB 153 HAA medium (Peptide Institute Inc) with 0.07 mM Ca^2+^, 5 mg/l insulin, 100 ng/l epidermal growth factor, 180 μg/l hydrocortisone, 6.1 mg/l monoethanolamine, 14.1 mg/l *O*-phosphorylethanolamine, and 0.4% (v/v) bovine pituitary extract. For differentiation, the culture medium was changed to a high Ca^2+^ medium (1.8 mM Ca^2+^ MCDB 153 HAA medium) after reaching confluence.

NHDFs (Detroit 551, CCL-110) were purchased from the American Type Culture Collection (Manassas). The cells were maintained in Eagle’s minimum essential medium (FUJIFILM Wako Pure Chemical) supplemented with non-essential amino acids, 1 mM sodium pyruvate, and 10% (v/v) FBS. The cells were cultured at 37 °C in a humidified atmosphere with 5% CO_2_.

For UVB irradiation, NHEKs were seeded in 24-well plates. After reaching confluence, the culture medium was removed and the cells were exposed to UVB using a UV Crosslinker (Ultra-Violet Products Ltd). Human recombinant IFN-γ was purchased from FUJIFILM Wako Pure Chemical.

### RNA interference

Knockdown experiments were performed using 2.5 nM negative control siRNA or target siRNAs. These siRNAs were transfected into cells using HiPerFect transfection reagent (Qiagen). After 24 h, the medium was replaced with fresh medium. The siRNA oligonucleotide sequences were as follows (Qiagen):

*HAS2* siRNA: 5′-CCUCAGCAGUGUAAGAUAU-3′

*HAS3* siRNA: 5′-CCAUCGAGAUGCUUCGAGU-3′

*HYAL1* siRNA: 5′-CCAUGUCCAUAUGCAUCUA-3′

*HYAL2* siRNA: 5′-CUGCCAGUACCUCAAAGAUUA-3′

*TMEM2* siRNA: 5′-CAGGAUGCUGGAAUAUGGUAUUUAU-3′

Negative control siRNA (Thermo Fisher Scientific).

### RT-qPCR

The cells were subjected to total RNA extraction using NucleoSpin RNA kit (Takara Bio Inc). Complementary DNA was prepared using PrimeScript RT Master Mix enzyme (Takara Bio Inc) in a reaction conducted at 37 °C for 15 min. PCR conditions were the same as reported previously (55). Briefly, 45 cycles were conducted under the following conditions: denaturation for 5 s at 95 °C, followed by annealing and extension for 30 s at 60 °C, using a CFX96 Deep Well Real-Time system (Bio-Rad Laboratories). *ATP5F1* was used as the reference gene for normalization.

The PCR primer sequences were:

*HYBID*-forward: 5′-GGCTTCTGAGCCGGAACATC-3′

*HYBID*-reverse: 5′-GCTGCCTTAAATCCCAGAGCAA-3′

*HYAL1*-forward: 5′-CCCTGCAGGTCAATGTTTAACAGA-3′

*HYAL1*-reverse: 5′-CAGAGCTGAGAACAGGTTGCAAAG-3′

*HYAL2*-forward: 5′-ACCATGCACTCCCAGTCTACGTC-3′

*HYAL2*-reverse: 5′-TCGCCAATGGTAGAGATGAGGTC-3′

*HAS2*-forward: 5′-CCTTCAGAGCACTGGGACGA-3′

*HAS2*-reverse: 5′-AGATGAGGCTGGGTCAAGCATAG-3′

*HAS3*-forward: 5′-GCAAGCGCGAGGTCATGTA-3′

*HAS3*-reverse: 5′-TGGATCCAGCACAGTGTCAGAG-3′

*Keratin1*-forward: 5′-AGATCACTGCTGGCAGACATGG-3′

*Keratin1*-reverse: 5′-TGATGGACTGCTGCAAGTTGG-3′

*TMEM2*-forward: 5′-TCCACAGTACCAGCCTGTCGTC-3′

*TMEM2*-reverse: 5′-TGATGGATAGCAAAGGCCAACTC-3′

*ATP5F1*-forward: 5′-GAAGCAGGCTTCCATCCAACA-3′

*ATP5F1*-reverse: 5′-TCGTTCCCGGTAAGTAACTTCCAA-3′

### Immunoblotting analysis

For total cell lysate, NHEKs were lysed in RIPA buffer [50 mM-Tris-HCl buffer (pH 8.0), 150 mM-NaCl, 1%-Nonidet P40 substitute (w/v), 0.5%-sodium deoxycholate (w/v), 0.1%-SDS (w/v), 1.0% NP-40, and protease inhibitor cocktail] (FUJIFILM Wako Pure Chemical) and sonicated using a BIORUPTOR Ⅱ Type 6 (CosmoBio). The supernatant was collected after centrifugation at 4 °C for 3 min (7000*g*). The protein concentrations were measured using a DC protein assay kit (Bio-Rad Laboratories). The samples were separated using 10% SuperSepAce gels (FUJIFILM Wako Pure Chemical) and transferred to PVDF membranes (Merck). Non-specific binding was blocked at room temperature (15–25 °C) for 15 min with Block Ace (KAC). The membranes were incubated overnight at 4 °C with the following primary antibodies: mouse anti-HYAL1 (Santa Cruz Biotechnology, sc-101340, 1:150), rabbit anti-KIAA1199 (HYBID, Sigma-Aldrich, SAB2105467, 1:1000), rabbit anti-TMEM2 (Sigma Aldrich, SAB2105088, 1:500), rabbit anti-CathepsinD (Proteintech Inc, 21327-1-AP, 1:1000), mouse anti-Na⁺-K⁺-ATPase (Santa Cruz Biotechnology, sc-21712, 1:1000), and mouse anti-GAPDH (Santa Cruz Inc, sc-47724, 1:2000). The primary antibodies were detected using HRP-conjugated secondary antibodies (GE Healthcare). Finally, the signals were visualized using ImmunoStar LD (FUJIFILM Wako Pure Chemical), and the expression level of each band was determined by quantifying the intensity using Amersham Imager 680 Analysis software (GE Healthcare).

### HA depolymerization assay for living cells using gel permeation chromatography analysis

After reaching confluence in a 24-well plate, a final concentration of 10 μg/ml FITC-HMW-HA (FA-HA H2; 1200–1600 kDa; PG Research, Tokyo, Japan) was added to the culture medium. After 48 h culture, the culture medium was collected and applied to a Sepharose CL-2B (GE Healthcare Bioscience) column (0.7 × 50 cm) equilibrated with 0.5 M NaCl. The flow rate was 0.15 ml/min, and 0.3 ml fractions were collected. The fluorescence of each fraction was measured using a GloMax-Multi Detection System (Ex. 490 nm/Em. 510–570 nm, Promega, Madison). The column was calibrated using FA-HA H2 and FA-HA L2 (100–300 kDa).

### Assay for extracellular HA degradation *in vitrovia* gel permeation chromatography

The cells were cultured in 24-well plates and reached confluence. The medium was replaced with culture or differentiation medium. For extracellular HA degradation assay *in vitro*, the culture medium was collected at various time points. The collected medium was then incubated at 37 °C for 12 h with 10 μg/ml FITC-HMW-HA H2 (1200–1600 kDa) and 20 μg/ml HA (1500–1700 kDa, Sigma-Aldrich) using acetate buffer to adjust the pH (pH 3.0–7.5). Subsequently, the reaction mixtures were transferred to the Sepharose CL-2B column (0.7 × 30 cm). The flow rate was maintained at 0.15 ml/min, and 0.3 ml fractions were collected. Subsequently, fluorescence was detected using a GloMax-Multi Detection System.

### Quantification of HA in the culture medium and determination of molecular size distribution

HA in culture medium or each fraction for MW distribution was determined using an HA Quantification Kit (PG Research) according to the manufacturer’s protocol. The culture medium was collected 24 h after changing the medium. The fractions of medium were collected using the Sepharose CL-2B column (0.7 × 50 cm) with a 0.15 ml/min flow rate and 0.3 ml fractions.

### Subcellular fraction

The cells were homogenized in ice-cold 50 mM Tris-HCl buffer (pH 7.5) with a protease inhibitor cocktail (FUJIFILM Wako Pure Chemical) using a potter-elvehjem homogenizer. The nuclear fraction (N) is the sediment from centrifuging at 500*g* for 3 min. The heavy mitochondrial fraction (M) is the sediment from centrifuging fraction N at 5000*g* for 3 min. The light mitochondrial fraction (L) is the sediment from centrifuging fraction M at 15,000*g* for 15 min. The microsomal fraction (P) is the sediment from centrifuging fraction L at 100,000*g* for 30 min. The cytosolic fraction (S) is the supernatant from centrifuging fraction L at 1,000,00*g* for 30 min.

### Statistical analysis

For statistical analysis of the results of RT-qPCR, immunoblotting assays, and HA quantification, the mean ± standard deviation (SD) was calculated for normalized distributions from different culture wells. Comparisons between multiple variables were calculated using Dunnett’s test and Student’s *t* test. Statistical analysis was performed using SPSS software (ver. 24.0, IBM). *p* < 0.05 was considered statistically significant. Varying statistical significance is indicated by: ∗*p* < 0.05; ∗∗*p* < 0.01; ∗∗∗*p* < 0.005. All experiments were performed at least thrice.

## Data availability

The data that support the findings of this study are available from the corresponding author [mizutani@gifu-pu.ac.jp] upon reasonable request.

## Supporting information

This article contains supporting information (61, 62).

## Conflict of interest

The authors declare that they have no conflicts of interest with the contents of this article.

## Supporting information

Table S1

Supporting Experimental procedures
