# Supplementary material for: Epidermal keratinocytes regulate hyaluronan metabolism via extracellularly secreted hyaluronidase 1 and hyaluronan synthase 3
Source: J Biol Chem. 2024 Jun 4;300(7):107449. doi: 10.1016/j.jbc.2024.107449 (PMC11292368; doi:10.1016/j.jbc.2024.107449)
Supplement: Table S1 [file mmc1.docx]

**TableS1. The transcripts per million (TPM) of HYAL1, HYAL2, HYBID, TMEM2, CD44, HAS1, HAS2, HAS3, ATP5F1, and GAPDH GAPDH in NHEK and NHDF.**

NHEK and NHFD were seeded in a 24-well plate and transfected with non-targeting control siRNA, and the medium was changed after 24h. After 24h of incubation, total RNA was collected, and a library labeled with a barcode sequence was prepared. The library was analyzed on a NextSeq 500. ATP5F1, β-ACTIN, GAPDH, and GUSB are shown as housekeeping genes. TPMs were calculated from gene length and the read count using the RNA-Seq analysis tool in the Genomic Workbench. Values represent the average±S.D.(n=3).

**NHEK NHDF NHEK/NHDF (fold)**

**HYAL1 4.75±0.72 0.5±0.54 9.50**

**HYAL2 67.46±8.03 49.96±1.84 1.35**

**HYBID (CEMIP) 8.23±1.50 356.02±61.10 0.02**

**TMEM2 (CEMIP2) 62.11±4.81 48.28±2.43 1.29**

**CD44 583.9±19.72 893.45±59.49 0.65**

**HAS1 1.39±0.59 0.14±0.12 9.93**

**HAS2 2.66±0.66 6.56±1.41 0.41**

**HAS3 60.42±4.90 0.76±0.18 79.50**

**ATP5F1 65.48±4.85 112.19±6.73 0.58**

**GAPDH 1978.96±339.84 4174.45±162.95 0.47**
