# Supplementary material for: Epidermal keratinocytes regulate hyaluronan metabolism via extracellularly secreted hyaluronidase 1 and hyaluronan synthase 3
Source: J Biol Chem. 2024 Jun 4;300(7):107449. doi: 10.1016/j.jbc.2024.107449 (PMC11292368; doi:10.1016/j.jbc.2024.107449)
Supplement: Supporting Experimental procedures [file mmc2.docx]

**Supporting Experimental procedures**

**Next-generation sequencing**

RNA was extracted from NHEK and NHDF using the RNeasy mini kit (QIAGEN) and was confirmed to be over 7.0 RIN using a Bioanalyzer. Poly (A) RNA was extracted from 100 ng of total RNA with an NEBNext poly(A) mRNA magnetic isolation module (NEB). The library was prepared according to the instruction manual of NEBNext ultra Ⅱ RNA library prep kit (NEB) (61, 62). Briefly, cDNA was synthesized and amplified by PCR to prepare a library labeled with a barcode sequence to identify the sample. The library was analyzed using a NextSeq500 (Illumina) with a 75 bp paired-end cycle sequencing kit (Illumina). The gene reads were mapped to the reference sequence following trimming, and tag counts were performed. The number of transcript reads corresponding to each gene ID and transcript ID were counted using the RNA-Seq Analysis tool (CLC Genomics Workbench), and normalized values (TPM, transcripts per million) were calculated based on the gene length and read count.

61. Kohno, M., Kobayashi, S., Yamamoto, T., Yoshitomi, R., Kajii, T., Fujii, S., Nakamura, Y., Kato, T., Uchinoumi, H., Oda, T., Okuda, S., Watanabe, K., Mizukami, Y., and Yano, M. (2020) Enhancing calmodulin binding to cardiac ryanodine receptor completely inhibits pressure-overload induced hypertrophic signaling. *Communications biology* **3**, 714

62. Muto, J., Fukuda, S., Watanabe, K., Dai, X., Tsuda, T., Kiyoi, T., Kameda, K., Kawakami, R., Mori, H., Shiraishi, K., Murakami, M., Imamura, T., Higashiyama, S., Fujisawa, Y., Mizukami, Y., and Sayama, K. (2023) Highly concentrated trehalose induces prohealing senescence-like state in fibroblasts via CDKN1A/p21. *Communications biology* **6**, 13
