# Supplementary material for: Epidermal keratinocytes regulate hyaluronan metabolism via extracellularly secreted hyaluronidase 1 and hyaluronan synthase 3
Source: J Biol Chem. 2024 Jun 4;300(7):107449. doi: 10.1016/j.jbc.2024.107449 (PMC11292368; doi:10.1016/j.jbc.2024.107449)
Supplement: Supporting information Figures [file mmc3.pdf]

Figure S1

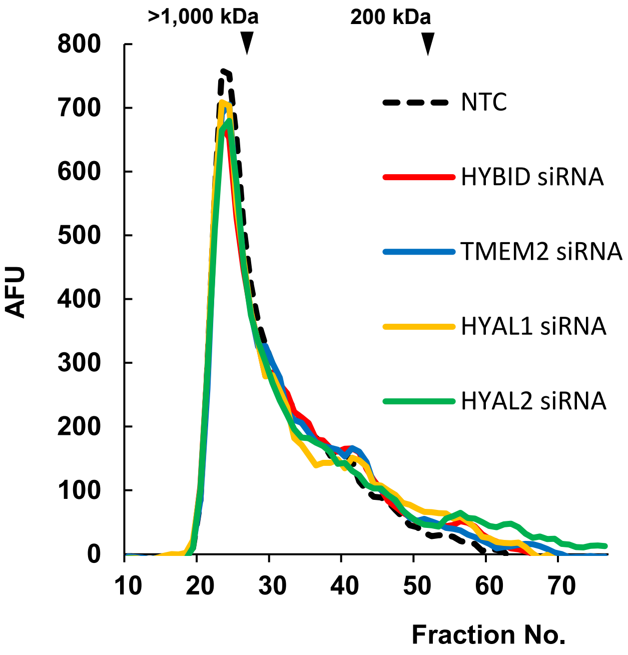


**Figure S1. The downregulation of candidate genes, *HYBID*, *TMEM2*, *HYAL1*, and *HYAL2*, is not related to the degradation of HMW-HA for living NHEKs**
Silenced NHEKs, NHEKs transfected with *HYBID* siRNA, *TMEM2* siRNA, *HYAL1* siRNA, *HYAL2* siRNA, or NTC were cultured with FITC-HMW-HA for 48 h. The culture media were collected, and the distribution of FITC-labeled HA was determined by the Sepharose CL-2B column (0.7 × 50 cm).

# Figure S2

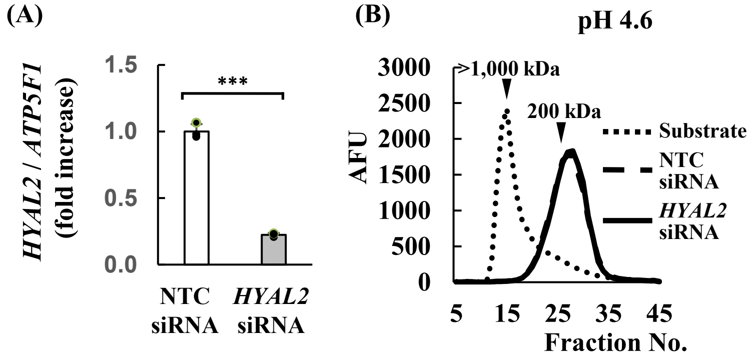


**Figure S2. HYAL2 is not related to HMW-HA degradation *in vitro***

(A) The mRNA expression of *HYAL2* following transfection with *HYAL2* siRNA was determined by RT-qPCR. The expression levels were normalized to *ATP5F1* used as a loading control. The expression of *HYAL2* in NTC siRNA-treated NHEK cells was set at 1.00, and the expression level of *HYAL2* siRNA was shown as a ratio. Values represent the mean ± S.D. (n = 3). ***p < 0.005 versus the control (NTC siRNA) (Student's t-test). (B) *In vitro* assay detected and performed at pH 4.6. The degradation of HA was separated by the Sepharose CL-2B column (0.7 × 30 cm).

Figure S3

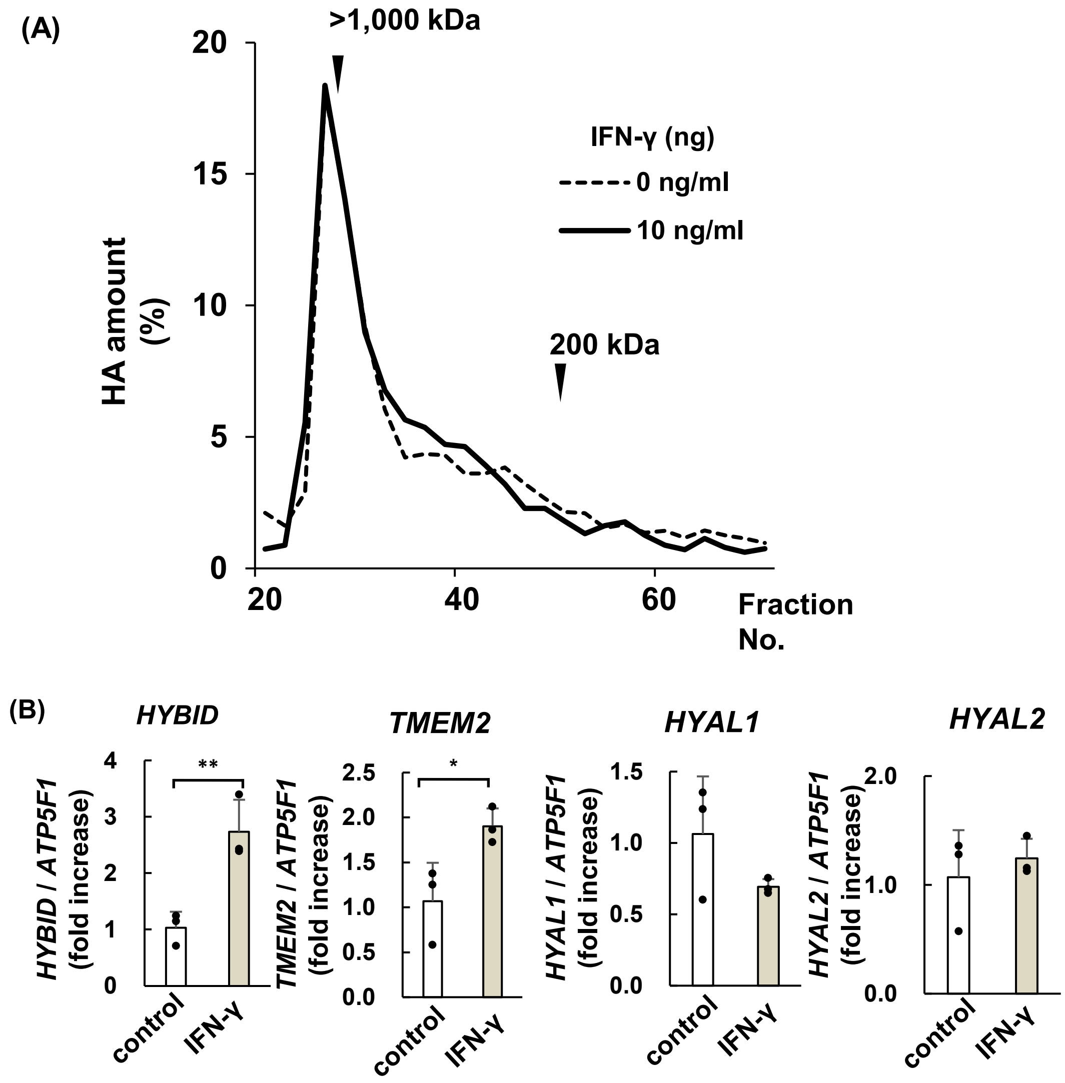


**Figure S3. IFN-γ did not change the molecular distribution of HA in NHEK cells**

(A) The % of HA in each fraction was calculated using the data from Fig. 6C. Molecular size distribution of HA after NHEK stimulation with or without IFN-γ (10 ng/ml) for 24 h. (B) mRNA levels of *HYBID*, *TMEM2*, *HYAL1*, and *HYAL2* following stimulation with IFN-γ (10 ng/ml) and culture for 6 h, detected by RT-qPCR. The expression level of the non-treated control was set to 1.0. *ATP5F1* was used as the loading control. Values represent the mean ± S.E.M. (n = 3) *p < 0.05, **p < 0.01 versus the control (UVB 0 mJ/cm$^2$) (Dunnett's test).

Figure S4

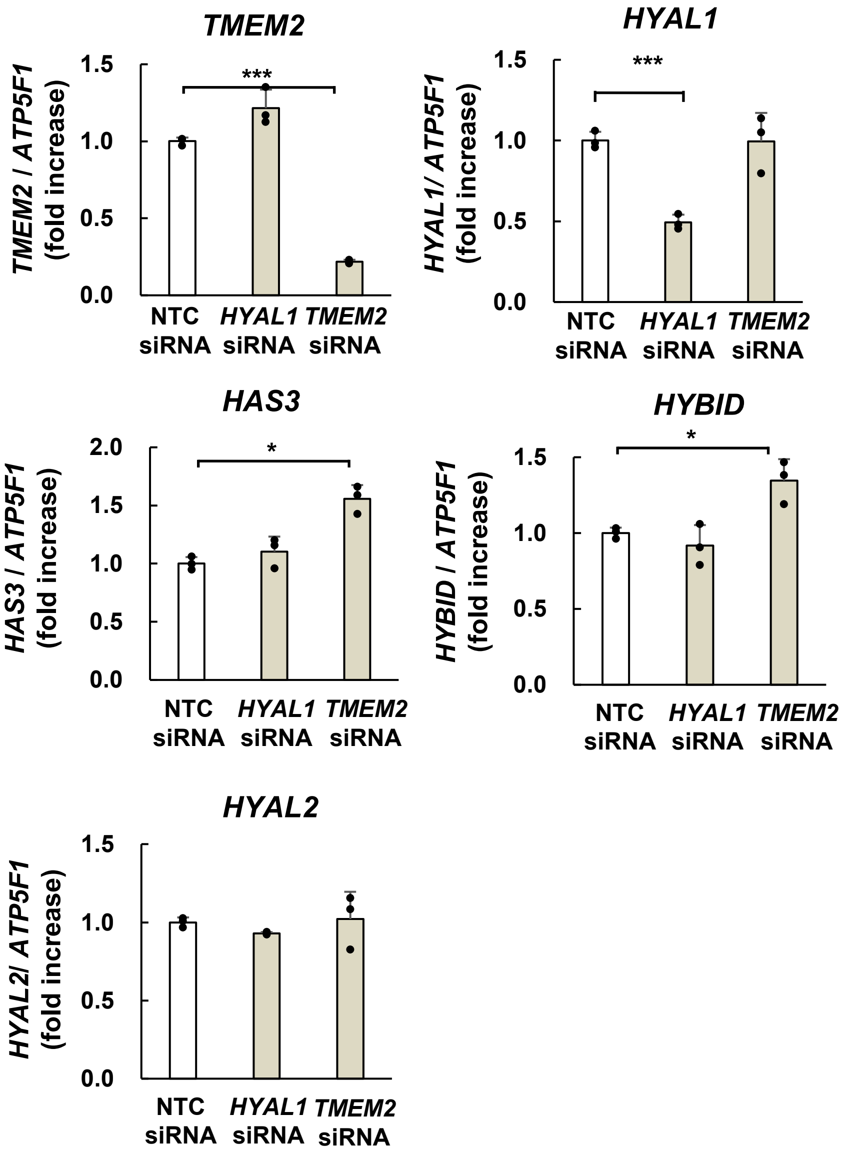


**Figure S4. The downregulation of *HYAL1* is not related to the expression of other HA metabolism-related genes, including *HAS3* mRNAs**

The mRNA levels of *TMEM2*, *HYAL1*, *HAS3*, *HYBID*, and *HYAL2* following transfection with *HYAL1* and *TMEM2* siRNA for 24 h were determined by RT-qPCR. The expression was normalized against that of *ATP5F1*, the loading control. The mRNA expression of NTC siRNA-treated NHEK cells was set at 1.00, and the expression following siRNA treatment is shown as a ratio. Values represent the mean ± S.D. (n = 3). ***p < 0.005 and *p < 0.05 versus the control (NTC siRNA) (Student's t-test).

# Figure S5

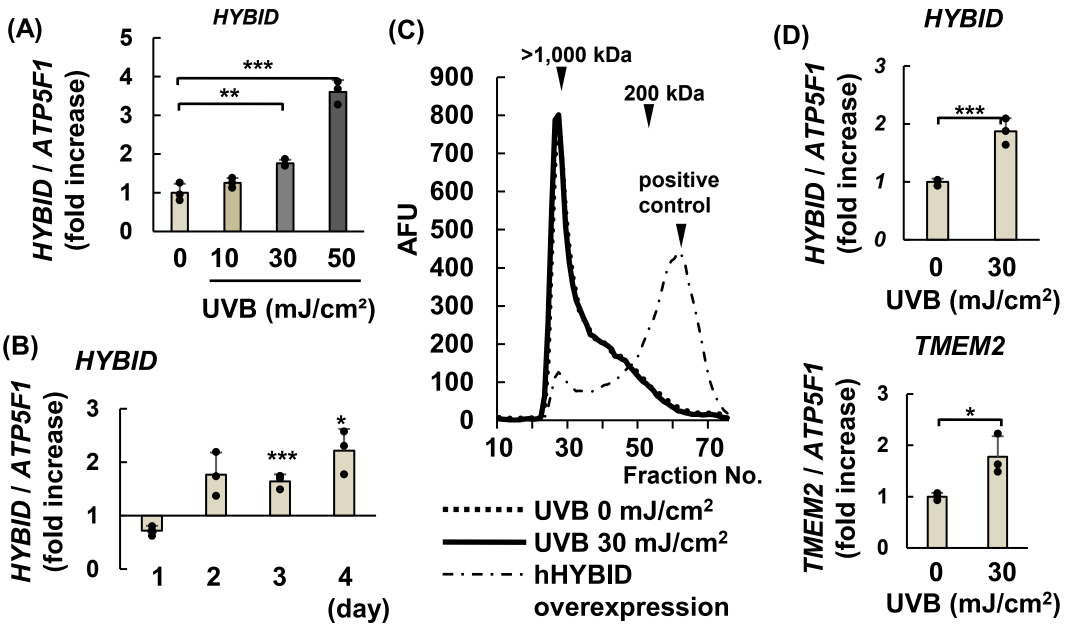


**Figure S5. HYBID is not the major molecule acting in the depolymerization of HMW-HA in NHEKs**

(A) Dose-dependent increase in *HYBID* mRNA expression by UVB (0, 10, 30, 50 mJ/cm$^2$). Two days post-irradiation, total RNA was prepared from NHEK cells and used for RT-qPCR. (B) Time-dependent increase in *HYBID* mRNA expression by UVB irradiation (30 mJ/cm$^2$). After irradiation, NHEKs were cultured in high-$Ca^{2+}$ medium for 1, 2, 3, and 4 d, and total RNA was collected for RT-qPCR. Values represent the mean ± S.D. (n = 3) *p < 0.05, **p < 0.01, ***p < 0.005 versus the control (UVB 0 mJ/cm$^2$) (Dunnett's test). (C) Cellular HA depolymerization in NHEK with or without UVB irradiation. One day after irradiation, NHEK cells were changed to high-$Ca^{2+}$ medium with FITC-HMW-HA for 2 d. The distribution of FITC-HA was determined by the Sepharose CL-2B column (0.7 × 50 cm). HEK293T cells transfected with human HYBID were used as the positive control. The positive control revealed a peak shift to LMW-HA. (D) *HYBID* and *TMEM2* were detected by RT-qPCR. The expression level before UVB irradiation was set to 1.0. *ATP5F1* was used as the loading control. Values represent the mean ± S.D. (n = 3) *p < 0.05, **p < 0.01, ***p < 0.005 versus the control (UVB 0 mJ/cm$^2$) (Dunnett's test).
